# Synthesis of Methyl 4,6-Di-*O*-ethyl-α-d-glucopyranoside-Based Azacrown Ethers and Their Effects in Asymmetric Reactions

**DOI:** 10.3390/molecules26154668

**Published:** 2021-08-02

**Authors:** István Orbán, Bertalan Varga, Péter Bagi, László Hegedűs, Péter Bakó, Zsolt Rapi

**Affiliations:** Department of Organic Chemistry and Technology, Budapest University of Technology and Economics, H-1111 Budapest, Hungary; istvan.orban@edu.bme.hu (I.O.); varga.bertalan@edu.bme.hu (B.V.); bagi.peter@vbk.bme.hu (P.B.); hegedus.laszlo@vbk.bme.hu (L.H.); bako.peter@vbk.bme.hu (P.B.)

**Keywords:** crown ether, carbohydrates, enantioselectivity, asymmetric synthesis

## Abstract

Carbohydrate-based crown ethers have been reported to be able to generate asymmetric induction in certain reactions. Previously, it was proved that the monosaccharide unit, the anomeric substituent, and the sidearm could influence the catalytic activity of the monoaza-15-crown-5 macrocycles derived from sugars. In order to gain information about the effect of the flexibility, 4,6-di-*O*-ethyl-glucoside-based crown compounds were synthesized, and their efficiency was compared to the 4,6-*O*-benzylidene analogues. It was found that the absence of the two-ring annulation has a negative effect on the enantioselectivity in liquid-liquid two-phase reactions: in the Darzens condensation of 2-chloroacetophenone and in the epoxidation of chalcone. The same trend was observed in the solid-liquid phase Michael addition of diethyl acetamidomalonate. Surprisingly, in the solid-liquid phase cyclopropanation of benzylidenemalononitrile, one of the new catalysts was highly enantioselective (99% ee).

## 1. Introduction

Catalysis is one of the key technologies for achieving sustainable chemistry. Organocatalysis is a recently developed method that is a particularly favorite field in enantioselective synthesis [1,2,3,4]. This technique uses metal-free compounds that can catalyze organic reactions. Asymmetric organocatalysis applies chiral organic molecules to access enantioenriched products. Within the extremely fast-growing field of organocatalysis, catalysts derived from chincona alkaloids have been successfully used in enantioselective syntheses [5,6,7,8,9,10,11,12,13,14]. The quaternization of the nitrogen of the quinuclidine unit in the cinchona alkaloids results in the formation of organocatalysts, which can also act as phase transfer catalysts.

Phase transfer catalysis (PTC), a special case of organocatalysis, is a useful technique in organic syntheses that has long been recognized as a simple method using mild reaction conditions, inexpensive and environmentally benign reagents, and solvents. PTC offers the possibility of realizing preparations on a larger scale, as well [15,16]. Since the 1990s, asymmetric PTC has emerged as a topic of scientific interest. Consequently, different chiral catalysts derived from cinchona alkaloids, amino acids, and 1,1′-bi-2-naphthol (BINOL) alongside macrocyclic compounds have been synthesized and applied [17,18,19,20,21,22,23,24,25,26,27,28,29,30,31,32,33,34,35].

Chiral crown ethers applied as phase transfer catalysts can be used in asymmetric syntheses. Cram and his coworkers have published the first enantioselective phase-transfer reaction using a chiral crown ether catalyst derived from synthetic 1,1′-bi-2-naphthol [36]. The development of chiral catalysts from naturally occurring and cheap enantiopure compounds (e.g., from carbohydrates) is one of the challenges of green chemistry.

Previously, it has been proven that crown ethers containing a monosaccharide unit annulated to the macro ring can generate asymmetric induction as chiral phase transfer catalysts in certain reactions [37,38,39]. Crown ethers incorporating sugar moieties have been synthesized from various carbohydrates, e.g., from d-glucose [40,41], d-galactose [42,43], d-mannose [44,45], d-altrose [46], d- and l-xylose [47,48], l-arabinose [49], d-mannitol [50,51,52] or l-threitol [53]. Thus, a broad scale of carbohydrate-based macrocycles with diverse chirality are available. A part of these crown ethers may be used as chiral phase transfer catalysts in asymmetric syntheses [54,55].

While the structure-activity relationship has been investigated in our research group, it was found that the most active catalysts are the monoaza-15-crown-5-type lariat ethers containing a monosaccharide unit. There are other moieties that affect the catalytic activity of the azacrown compounds in addition to the carbohydrate unit, such as the sidearm on the nitrogen [56], the substituent and the configuration of the anomeric center [57,58,59], and the acetal group in the 4,6-position of the monosaccharide [60,61].

The bicyclic acetal structure provides rigidity to the carbohydrate moiety. Thus, the conformational changes require more energy, which may have a crucial role in enantioselectivity. Investigation of azacrown ethers bearing more flexible butyl substituents in the 4,6 position of the glucose molecule revealed that the effects of the individual moieties are not independent. Application of a crown catalyst having larger conformational freedom can lead to higher enantioselectivity depending on the structure of the sidearm [62].

**Figure 1:**
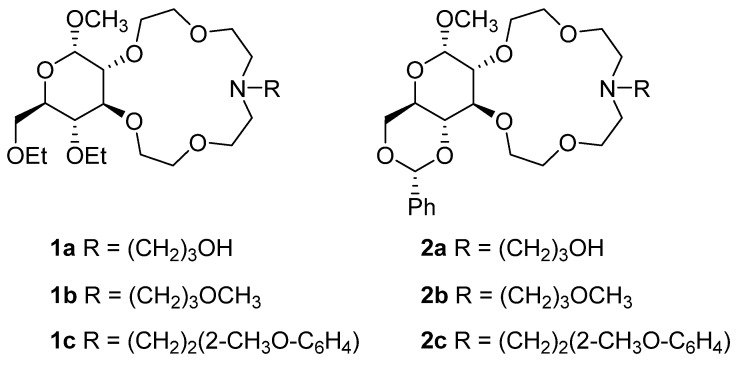
Crown ethers derived from 4,6-di-*O*-ethyl-glucoside (**1a**–**c**) and their 4,6-*O*-benzylidene analogues (**2a**–**c**).

Herein, we report the synthesis of a few methyl 4,6-di-*O*-ethyl-glucoside-based crown ethers bearing different side chains on the nitrogen. The most effective sidearms to date, hydroxypropyl (**1a**), methoxypropyl (**1b**), and 2-methoxyphenylethyl groups (**1c**) were incorporated into the new catalysts (Figure 1). Ethyl groups provide greater flexibility to crown compounds **1a**–**c**; however, they do not excessively increase lipophilicity. The efficiency of the macrocycles **1a**–**c** was investigated in different asymmetric reactions, and the results were compared with those obtained with their benzylidene analogues **2a**–**c** (Figure 1) to establish correlations between the effect and the structure. Crown ether **2a** was previously investigated in all the model reactions applied for the catalyst testing [63,64,65,66].

## 2. Results and Discussion

### 2.1. Synthesis of Azacrown Ethers

**Scheme 1:**
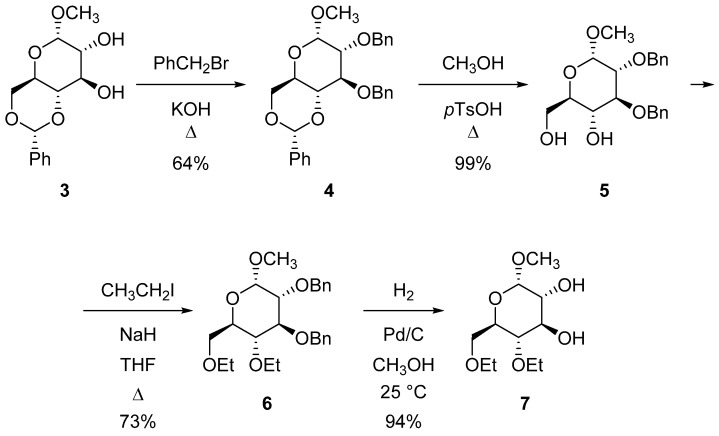
Synthesis of methyl 4,6-di-*O*-ethyl-α-d-glucopyranoside (**7**) from methyl 4,6-*O*-benzylidene-α-d-glucopyranoside (**3**).

According to the literature, the starting material, methyl 4,6-*O*-benzylidene-α-d-glucopyranoside (**3**), was previously synthesized in our research group [67]. Next, the free 2- and 3-hydroxy groups were benzylated with benzyl bromide using a solid potassium hydroxide base in boiling toluene as stated in a previously reported method (Scheme 1) [68]. Full conversion was achieved after 6 h, and the pure dibenzyl product **4** was obtained after recrystallization from ethanol with a good yield.

Subsequently, the benzylidene group of compound **4** was removed by transacetalation. Excess methanol was used as both reagent and solvent in the reaction, and para-toluenesulfonic acid acted as a catalyst (Scheme 1) [69]. Under anhydrous conditions, the benzylidene group is cleaved by the formation of benzaldehyde dimethyl acetal, liberating the 4- and 6-hydroxy groups in derivative **5**. After the workup procedure, the crude product was a colorless syrup, from which fibrous crystals precipitated upon cooling in a refrigerator. The white, fluffy product **5** was obtained in almost quantitative yield after washing with hexane.

Afterward, the free 4- and 6-hydroxy groups of the diol **5** were alkylated with ethyl iodide in the presence of sodium hydride in anhydrous tetrahydrofuran under argon (Scheme 1). To achieve complete conversion, a large excess of both the base and the alkylating agent had to be applied, and a total of 40 h of boiling was required. The most abundant impurity was the monoalkylated derivative, from which the main product **6** was purified by column chromatography.

The last step before the construction of the crown ring was the removal of benzyl groups from the 2- and 3-positions of glucoside **6**. Selective deprotection was carried out by catalytic hydrogenation in an autoclave (15 bar hydrogen pressure) in the presence of Pd/C (Scheme 1). The ^1^H and ^13^C NMR spectra of the glucose derivative **7** showed the absence of the aromatic and benzylic signals, thus confirming the completion of the reaction.

**Scheme 2:**
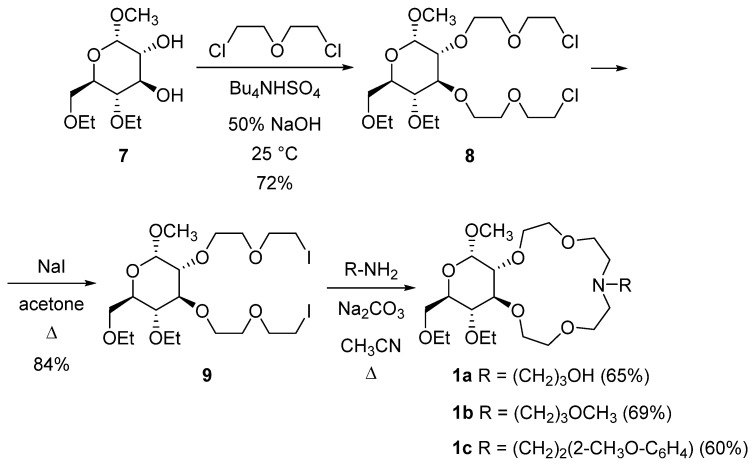
Preparation of azacrown ethers **1a**–**c** in three steps.

As described previously by us in many cases, the crown structure was synthesized in three steps [55]. The free vicinal hydroxyl groups of compound **7** were alkylated with bis(2-chloroethyl) ether in a liquid-liquid two-phase system, in which Bu_4_OH was generated from 50% aq. NaOH and tetrabutylammonium hydrogensulfate (Scheme 2). After chromatography, bischloro podand **8** was obtained in good yield (72%). The exchange of chlorine to iodine was performed by reacting bischloro compound **8** with NaI in dry acetone (Scheme 2). Derivative **9** was isolated without purification in a yield of 84%. Macrocyclization was carried out with 3-aminopropanol, 3-methoxypropylamine, and 2-(2-methoxyphenyl)ethylamine in boiling acetonitrile, applying Na_2_CO_3_ as the base (and to exploit the template effect) to provide azacrown ethers **1a**–**c** in yields of 60–69% after column chromatography (Scheme 2).

Several attempts were made to prepare catalysts **1a**–**c** in a more concise way, starting from crown compounds **2a**–**c**. Removal of the benzylidene group proceeded easily; however, the alkylation of the crown ethers bearing free OH groups gave the expected products in relatively low yields (<10%). Presumably, side reactions were initiated with the alkylation of the nitrogen, resulting in a mixture of inseparable compounds.

### 2.2. Enantioselective Reactions

Crown ethers **1a**–**c** derived from 4,6-di-*O*-ethyl-glucopyranoside were tested in two asymmetric liquid-liquid and two solid-liquid phase transfer reactions. The results were compared to the effects of the analogous 4,6-*O*-benzylidine catalysts **2a**–**c**. The results of the asymmetric reactions in the presence of crown ether **2a** have already been reported in previous works [63,64,65,66]. In all cases, 10 mol% of the crown compound (**1** or **2**) was used. After completion of the reaction, crude products were isolated by preparative thin-layer chromatography (TLC). Chiral HPLC measurements determined the ee values. In each asymmetric reaction, it was always the same enantiomers that were formed in excess.

**Scheme 3:**
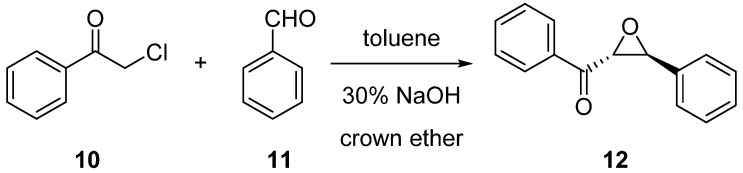
Darzens condensation of 2-chloroacetophenone (**10**) and benzaldehyde (**11**) in the presence of sugar-based crown ethers (**1** or **2**).

One of the asymmetric reactions was the base-initiated Darzens condensation of 2-chloroacetophenone (**10**) and benzaldehyde (**11**) (Scheme 3). The synthesis resulted in chiral epoxide **12** with complete diastereoselectivity, while a new C-C bond was established. The absolute configuration of epoxyketone **12** was previously assigned as 2*R*,3*S* [63,70]. Using chiral crown ethers **1a**–**c**, full conversion was reached within one hour, as was the case with benzylidene analogues **2a**–**c** (Table 1). The same trend was observed for both series of macrocycles. The best enantioselectivity was provided by catalysts **1a** and **2a** bearing a hydroxypropyl sidearm (52% and 62%, Table 1, entries 1 and 4). When a methoxypropyl group was present, the asymmetric induction decreased to 29% (**1b**) and 21% (**2b**), respectively (Table 1, entries 2 and 5).

Previously, in liquid-liquid phase transfer reactions, the hydroxypropyl side-chain proved to be more effective than the methoxypropyl group in all cases [55]; this trend has persisted. Macrocycles with a methoxyphenylethyl substituent (**1c** and **2c**) also generated low enantiomeric excess values (19% and 29%, Table 1, entries 3 and 6). Comparing the data shows that the presence of a lipophilic side-chain negatively affects the catalytic activity in the Darzens condensation. Replacement of the benzylidene unit of catalysts **2a**–**c** with ethyl groups resulted in similar catalytic activity. While using crown ether **2a** gave the best ee value (62%, Table 1, entry 4), its **1a** analogue generated somewhat lower enantioselectivity (52%, Table 1, entry 1) was the highest among the diethyl substituted macrocycles. It can be concluded that the rigidity is not the most crucial property of the carbohydrate-based crown ethers in the Darzens reaction.

**Scheme 4:**
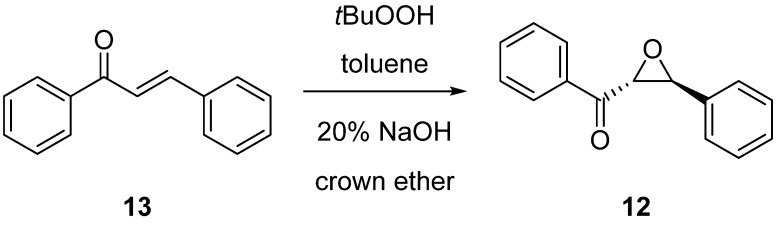
Epoxidation of chalcone (**13**) with *tert*-butyl hydroperoxide in the presence of sugar-based crown ethers (**1** or **2**).

Chiral epoxyketone **12** was also synthesized by epoxidation of *trans*-chalcone (**13**) under basic conditions (Scheme 4). Applying the crown catalysts, the highest enantioselectivity was again generated by **1a** and **2a** having a hydroxypropyl substituent (75% ee and 92% ee, respectively; Table 2, entries 1 and 4). The same phenomenon was experienced as before, i.e., when a methoxypropyl group was attached to the nitrogen, ee values were low with macrocycles **1b** and **2b** (24% ee and 23% ee, Table 2, entries 2 and 5). Crown ether **2c** bearing a methoxyphenylethyl group proved to be ineffective (72 h, 3% ee, Table 2, entry 6), while interestingly, its **1c** analogue generated low but significantly higher enantiomeric excess in a shorter time (24 h, 21%, Table 2, entry 3). In the case of **1a** and **1b**, elongation of the reaction time was experienced (4 h for both, Table 2, entries 1 and 2) compared to catalysts **2a** and **2b** (1 h and 2 h, respectively, Table 2, entries 4 and 5).

It can be concluded that the less rigid diethyl-substituted crown compounds (**1a**–**c**) showed lower efficiency in the epoxidation reaction than catalysts **2a**–**c**, having a benzylidene protecting group. However, comparing the results obtained with catalysts **1c** and **2c**, it can be seen that the effect of the side chain and that of the protecting group on the enantioselectivity are not independent of each other.

**Scheme 5:**
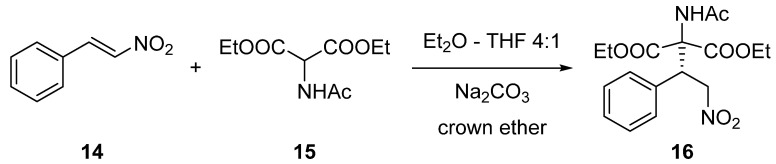
Michael addition of diethyl acetamidomalonate (**15**) to β-nitrostyrene (**14**) the presence of sugar-based crown ethers (**1** or **2**).

Asymmetric Michael addition offers an efficient method to prepare various products with new C-C bonds using electron-deficient olefins and CH-acidic compounds. The reaction of β-nitrostyrene (**14**) and diethyl acetamidomalonate (**15**) was investigated previously in our research group (Scheme 5). It has been found that using diethyl ether and THF in a ratio of 4:1 as the solvent significantly increases the enantiomeric excess generated by the sugar-based crown ether **2a** (99% ee, Table 3, entry 4) [66]. Its diethyl analogue **1a**, however, showed only modest enantioselectivity under the same conditions (42% ee, Table 3, entry 1). The absolute configuration of compound **16** was previously reported to be *S* [71].

Asymmetric induction decreased again when a methoxypropyl sidearm was present (**1b**: 28% ee, **2b**: 38% ee, Table 3, entries 2 and 5). Even lower ee values (21% and 15%, respectively, Table 3, entries 3 and 6) and significantly longer reaction times (120 h) were measured in the case of methoxyphenylethyl-substituted crown compounds **1c** and **2c**.

Again, the replacement of the benzylidene moiety led to increased reaction times and lower asymmetric induction. The most striking difference was observed between lariat ether **1a** and **2a** when a highly enantioselective catalyst was converted into a less effective one with the change of the protecting group.

**Scheme 6:**
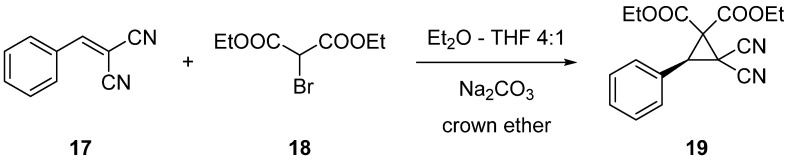
Cyclopropanation reaction of benzylidenemalononitrile (**17**) with diethyl bromomalonate (**18**) in the presence of glucose-based catalysts (**1** or **2**).

Finally, an enantioselective cyclopropanation reaction was investigated, in which two new C-C bonds were formed in two steps. The first step is a Michael addition, followed by an intramolecular cyclization, while a leaving group is detached. Because of this mechanism, this reaction is called the Michael-initiated ring-closure (MIRC) reaction. In our model reaction, benzylidenemalononitrile (**17**) served as the Michael acceptor, and diethyl bromomalonate (**18**) was the CH-acidic compound possessing a leaving group (Scheme 6). The absolute configuration of cyclopropane derivative **19** was previously assigned as *R* [72].

As shown in Table 4, in this reaction, catalysts **1a** and **2a** showed only low enantioselective catalytic activity (22% and 32%, respectively, Table 4, entries 1 and 4). The presence of a methoxypropyl side-chain significantly increased the asymmetric induction. While in the case of crown ether **2b**, compound **19** was isolated with excellent yield (97%) and good enantiomeric excess (70% ee) (Table 4, entry 5), the diethyl analogue **1b** proved to be highly enantioselective in this reaction (99% ee, Table 4, entry 2), however, the yield of cyclopropane derivative **19** was only moderate (40%).

There was a major difference between the effect of macrocycles **1c** and **2c**. Application of the former one (**1c**) led to a weak result (15% ee, Table 4, entry 3), while in the presence of **2c** an ee of 58% was observed (Table 4, entry 6). Again, these results strongly suggest that the side chain and the protecting group do not affect the enantioselectivity independently. With increased flexibility, catalysts **1c** showed weaker result (15% ee, Table 4, entry 3) than crown ether **2c** (58% ee, Table 4, entry 6), while the less rigid lariat ether **1b** was superior to macrocycle **2b** (99% ee and, 70% ee, respectively, Table 4, entries 2 and 5).

## 3. Materials and Methods

### 3.1. General

Chemicals were purchased from Merck KGaA. Analytical and preparative thin-layer chromatography was performed on silica gel plates (60 GF-254, Merck, Kenilworth, NJ, USA), while column chromatography was carried out using 70–230 mesh silica gel and Brockman-II neutral aluminum oxide. Visualization of compounds on the TLC plates was performed using 254 nm UV light, iodine or 5 *v*/*v*% sulfuric acid/methanol stain. Melting points were determined using a Stuart SMP10 apparatus and are uncorrected. The specific rotation was measured on a Perkin-Elmer 341LC polarimeter at 22 °C and 589 nm. NMR spectra were obtained on a Bruker DRX-500 or Bruker-300 instrument in CDCl_3_ with Me_4_Si as an internal standard. HRMS measurements were performed using Q-TOF Premier mass spectrometer (Waters, Milford, MA, USA) in positive electrospray ionization mode. The enantiomeric excess values were determined on a PerkinElmer Series 200 liquid chromatography system using different columns. In all cases, isocratic elution was applied with a mobile phase flow rate of 0.8 mL/min. The temperature was 20 °C, and the wavelength of the detector was 254 nm.

### 3.2. Synthesis of Crown Ethers

#### 3.2.1. Methyl-2,3-di-*O*-benzyl-4,6-di-*O*-ethyl-α-d-glucopyranoside (**6**)

Methyl-2,3-di-*O*-benzyl-α-d-glucopyranoside (**5**) (19.2 g, 51.3 mmol) was dissolved in dry tetrahydrofurane (200 mL) under argon atmosphere, and sodium hydride (3.67 g, 152.9 mmol) was added in small portions. The mixture was heated to reflux, and ethyl iodide (31.8 g, 203.9 mmol) was added dropwise. TLC showed incomplete conversion after 30 h of reflux; thus, surplus reagents (2.5 g, 104.2 mmol sodium hydride; 31.8 g, 203.9 mmol ethyl iodide) were added and the mixture was refluxed for another 10 h, after which conversion was complete. The reaction was quenched by dropwise addition of water (10 mL), and the mixture was concentrated in vacuum. The crude material was dissolved in a mixture of dichloromethane (100 mL), water (40 mL), and the phases were separated. The aqueous phase was extracted with dichloromethane (2 × 30 mL), the extracts were combined with the organic phase, and this was washed with water (150 mL), dried (Na_2_SO_4_), filtered, and concentrated in vacuum. The crude product was purified by column chromatography on a bed of silica gel (350 g) with hexane-ethyl acetate 3:2. Yield: 73% (16.05 g), yellow, viscous oil. ${\left[\alpha \right]}_{D}^{25}$ = +71.6 (*c* = 1, CHCl_3_).

^1^H NMR (300 MHz, CDCl_3_) δ 7.43–7.23 (m, 10H, Ar*H*); 5.00–4.78 (m, 2H, ArC*H*_2_O); 4.86–4.60 (m, 2H, ArC*H*_2_O); 4.61 (d, *J* = 3.6 Hz, 1H, H-1); 3.96–3.78 (m, 2H, H-6a, H-5); 3.70–3.35 (m, 11H, H-2, H-4, H-3, H-6b, 2 x OC*H*_2_, OC*H*_3_); 1.22 (t, *J* = 7.0 Hz, 3H, CH_2_C*H*_3_); 1.19 (t, *J* = 7.0 Hz, 3H, CH_2_C*H*_3_); ^13^C NMR (300 MHz, CDCl_3_) δ 139.99 (Ar*C*); 138.29 (Ar*C*); 128.43 (Ar*C*); 128.37 (Ar*C*); 127.98 (Ar*C*); 127.87 (Ar*C*); 127.56 (Ar*C*); 98.29 (*C-1*); 82.07 (*C*H); 79.67 (*C*H); 77.68 (*C*H); 75.72 (*C*H); 73.44 (Ar*C*H_2_); 70.12 (Ar*C*H_2_); 68.87 (*C*H_2_CH_3_); 68.32 (*C*H_2_CH_3_); 66.86 (O*C*H_2_CH); 55.12 (O*C*H_3_); 15.83 (CH_2_*C*H_3_); 15.11 (CH_2_*C*H_3_).

HRMS calculated for C_25_H_34_O_6_ 430.2355, found 430.2360.

#### 3.2.2. Methyl-4,6-di-*O*-ethyl-α-d-glucopyranoside (**7**)

Methyl-2,3-di-*O*-benzyl-4,6-di-*O*-ethyl-α-d-glucopyranoside (**6**) (16.05 g, 37.3 mmol) was subjected to hydrogenolysis in an autoclave in 100 mL of methanol using Selcat Q-6 type Pd/C catalyst (1.60 g). After completion of the reaction, the mixture was filtered and concentrated. The crude product was dissolved in dichloromethane and filtered through fine filter paper to remove all traces of the catalyst. The resulting clear solution was concentrated in vacuum. Yield: 94% (8.78 g), light-brown solid. ${\left[\alpha \right]}_{D}^{25}$ = +27.1 (*c =* 1, CHCl_3_). Mp. 79–83 °C.

^1^H NMR (300 MHz, CDCl_3_) δ 4.79 (d, *J* = 3.8 Hz, 1H, H-1); 3.88–3.79 (m, 1H, H-6a); 3.75 (t, *J* = 9.3 Hz, 1H, H-5); 3.71–3.46 (m, 7H, H-3, H-4, H-6b, 2 x OC*H*_2_); 3.41 (s, 3H, OC*H*_3_); 3.34 (t, *J* = 9.3 Hz, 1H, H-2); 1.24 (t, *J* = 7.0 Hz, 3H, CH_2_C*H*_3_); 1.21 (t, *J* = 7.0 Hz, 3H, CH_2_C*H*_3_); (OH groups were not visible); ^13^C NMR (300 MHz, CDCl_3_) δ 99.14 (*C-1*); 77.40 (*C*H); 75.10 (*C*H); 72.58 (*C*H); 70.28 (*C*H); 68.89 (*C*H_2_CH_3_); 68.11 (*C*H_2_CH_3_); 66.93 (O*C*H_2_CH); 55.29 (O*C*H_3_); 15.76 (CH_2_*C*H_3_); 15.11 (CH_2_*C*H_3_).

HRMS calculated for C_11_H_22_O_6_ 250.1416 found 250.1422.

#### 3.2.3. Methyl-4,6-di-*O*-ethyl-2,3-bis-*O*-[(2-chloroethoxy)ethyl]-α-d-glucopyranoside (**8**)

A two-necked round-bottomed flask was fitted with a mechanical stirrer and was charged with methyl-4,6-di-*O*-ethyl-α-d-glucopyranoside (**7**) (8.78 g, 35.2 mmol) and bis(2-chloroethyl)ether (124 mL, 1.06 mol). To this solution was added tetrabutylammonium hydrogensulfate (12.00 g, 35.2 mmol) and 50 m/m% NaOH solution (124 mL; 94.6 g, 2.36 mol NaOH). The resulting mixture was stirred vigorously for 12 h, after which it was diluted with dichloromethane (250 mL) and water (250 mL). The phases were separated, the aqueous layer was extracted with dichloromethane (4 × 100 mL). The combined organic phase was washed with water (3 × 100 mL), dried over Na_2_SO_4,_ and concentrated on a rotary evaporator. The excess bis(2-chloroethyl)ether was removed by vacuum distillation, and the crude product (18.33 g) was purified by column chromatography on a bed of silica gel (370 g). Gradient elution was used, CHCl_3_ → CHCl_3_-MeOH 100:2. Yield: 72% (11.74 g), orange, viscous oil. ${\left[\alpha \right]}_{D}^{25}$ = +65.1 (*c* = 1, CHCl_3_).

^1^H NMR (300 MHz, CDCl_3_) δ 4.83 (d, *J* = 3.6 Hz, 1H, H-1); 4.04–3.96 (m, 1H, H-6a); 3.94–3.72 (m, 9H, H-3, H-5, H-6b, 3 x OC*H*_2_); 3.69–3.56 (m, 13H, H-4, 4 x OC*H*_2_, 2 x C*H*_2_Cl); 3.49 (dd, *J* = 9.4; 7.0 Hz, 1H, H-2); 3.43–3.34 (m, 5H, OC*H*_2_, OC*H*_3_); 1.22 (t, *J* = 7.0 Hz, 3H, CH_2_C*H*_3_); 1.20 (t, *J* = 7.0 Hz, 3H, CH_2_C*H*_3_); ^13^C NMR (300 MHz, CDCl_3_) δ 92.23 (*C-1*); 82.61 (*C*H); 81.09 (*C*H); 77.70 (*C*H); 72.54 (*C*H); 71.50 (O*C*H_2_CH_2_); 71.41 (O*C*H_2_CH_2_); 71.18 (O*C*H_2_CH_2_); 71.08 (O*C*H_2_CH_2_); 70.98 (O*C*H_2_CH_2_); 70.32 (O*C*H_2_CH_2_); 69.10 (*C*H_2_CH_3_); 68.47 (*C*H_2_CH_3_); 67.06 (O*C*H_2_CH); 55.21 (O*C*H_3_); 42.95 (CH_2_*C*H_2_Cl); 42.90 (CH_2_*C*H_2_Cl); 16.02 (CH_2_*C*H_3_); 15.31 (CH_2_*C*H_3_).

HRMS calculated for C_19_H_36_Cl_2_O_8_ 462.1718, found 462.1722.

#### 3.2.4. Methyl-4,6-di-*O*-ethyl-2,3-bis-*O*-[(2-iodoethoxy)ethyl]-α-d-glucopyranoside (**9**)

Methyl-4,6-di-*O*-ethyl-2,3-bis-*O*-[(2-chloroethoxy)ethyl]-α-d-glucopyranoside (**8**) (11.70 g, 25.3 mmol) was dissolved in dry acetone (150 mL). Sodium iodide (15.00 g, 100.1 mmol) was added, and the mixture was refluxed for 50 h, during which a white precipitate was formed. The reaction mixture was filtered and concentrated. The crude product was dissolved in dichloromethane and washed with water (4 × 40 mL), dried over Na_2_SO_4_, and concentrated again. Yield: 84% (13.77 g), brown, viscous oil. ${\left[\alpha \right]}_{D}^{25}$ = +49.5 (*c* = 1, CHCl_3_).

^1^H NMR (300 MHz, CDCl_3_) δ 4.82 (d, *J* = 3.5 Hz, 1H, H-1); 4.02–3.95 (m, 1H, H-6a); 3.92–3.82 (m, 3H, H-5, OC*H*_2_); 3.92–3.70 (m, 6H, H-3, H-6b, 2 x OC*H*_2_); 3.69–3.54 (m, 9H, H-4, 4 x OC*H*_2_); 3.53–3.43 (m, 1H, H-2); 3.43–3.33 (m, 5H, OC*H*_2_, OC*H*_3_); 3.30–3.20 (m, 4H, 2 x C*H*_2_I); 1.1 (t, *J* = 7.0 Hz, 3H, CH_2_C*H*_3_); 1.19 (t, *J* = 7.0 Hz, 3H, CH_2_C*H*_3_); ^13^C NMR (300 MHz, CDCl_3_) δ 98.25 (*C-1*); 82.63 (*C*H); 81.04 (*C*H); 77.70 (*C*H); 72.57 (*C*H); 72.12 (O*C*H_2_CH_2_); 72.07 (O*C*H_2_CH_2_); 71.12 (O*C*H_2_CH_2_); 70.78 (O*C*H_2_CH_2_); 70.59 (O*C*H_2_CH_2_); 70.33 (O*C*H_2_CH_2_); 69.09 (*C*H_2_CH_3_); 68.50 (*C*H_2_CH_3_); 67.08 (O*C*H_2_CH); 55.25 (O*C*H_3_); 16.08 (CH_2_*C*H_3_); 15.33 (CH_2_*C*H_3_); 3.10 (CH_2_*C*H_2_I); 3.01 (CH_2_*C*H_2_I).

HRMS calculated for C_19_H_36_I_2_O_8_ 646.0500, found 646.0507.

#### 3.2.5. Methyl-4,6-di-*O*-ethyl-2,3-dideoxy-α-d-glucopyranosido[2,3-h]-*N*-[3-hydroxypropyl]-1,4,7,10-tetraoxa-13-azacyclopentadecane (**1a**)

Methyl-4,6-di-*O*-ethyl-2,3-bis-*O*-[(2-iodoethoxy)ethyl]-α-d-glucopyranoside (**9**) (2.80 g, 4.33 mmol) was dissolved in dry acetonitrile (60 mL), then 3-aminopropanol (0.33 g, 4.33 mmol) and Na_2_CO_3_ (2.76 g, 26.0 mmol) were added. The mixture was refluxed under Ar atmosphere for 40 h. Upon completion of the reaction, the mixture was filtered, and the filtrate was concentrated. The crude product was dissolved in dichloromethane and washed with water (3 × 20 mL), then the aqueous phase was extracted with dichloromethane (2 × 20 mL), and the organic phases were combined. This combined organic phase was dried over Na_2_SO_4_ and concentrated in vacuum affording 1.75 g of crude product. This was purified by column chromatography on an aluminum-oxide bed (52.5 g). Gradient elution was used CH_2_Cl_2_ → CH_2_Cl_2_-MeOH 100:1.

Yield: 65% (1.30 g), yellow, viscous oil. ${\left[\alpha \right]}_{D}^{25}$ = +68.3 (*c* = 1, CHCl_3_).

^1^H NMR (300 MHz, CDCl_3_) δ 4.83 (d, *J* = 3.6 Hz, 1H, H-1); 4.15–4.06 (m, 1H, H-6a); 3.85–3.53 (m, 20H, H-3, H-4, H-5, H-6b, 7 x OC*H*_2_, C*H*_2_OH); 3.53–3.44 (m, 1H, H-2); 3.43–3.33 (m, 5H, OC*H*_3_, OC*H*_2_); 3.00–2.64 (m, 6H, 3 x NC*H*_2_); 1.75–1.67 (m, 2H, CH_2_C*H*_2_CH_2_); 1.22 (t, *J* = 7.0 Hz, 3H, CH_2_C*H*_3_); 1.18 (t, *J* = 7.0 Hz, 3H, CH_2_C*H*_3_); ^13^C NMR (300 MHz, CDCl_3_) δ 97.58 (*C-1*); 81.85 (*C*H); 80.28 (*C*H); 77.98 (*C*H); 72.79 (*C*H); 70.81 (2 x O*C*H_2_CH_2_); 70.38 (2 x O*C*H_2_CH_2_); 70.28 (2 x O*C*H_2_CH_2_); 69.12 (*C*H_2_CH_3_); 68.44 (*C*H_2_CH_3_); 67.11 (O*C*H_2_CH); 59.77 (*C*H_2_OH); 55.20 (O*C*H_3_); 54.70 (2xN*C*H_2_); 54.56 (N*C*H_2_); 29.94 (NCH_2_*C*H_2_CH_2_OH); 16.08 (CH_2_*C*H_3_); 15.36 (CH_2_*C*H_3_).

HRMS calculated for C_22_H_43_NO_9_ 465.2938, found 465.2940.

#### 3.2.6. Methyl-4,6-di-*O*-ethyl-2,3-dideoxy-α-d-glucopyranosido[2,3-h]-*N*-[3-methoxypropyl]-1,4,7,10-tetraoxa-13-azacyclopentadecane (**1b**)

Methyl-4,6-di-*O*-ethyl-2,3-bis-*O*-[(2-iodoethoxy)ethyl]-α-d-glucopyranoside (**9**) (2.80 g, 4.33 mmol) was dissolved in dry acetonitrile (60 mL), then 3-methoxypropylamine (0.39 g, 4.33 mmol) and Na_2_CO_3_ (2.76 g, 26.0 mmol) were added. The mixture was refluxed under Ar atmosphere for 40 h. Upon completion of the reaction, the mixture was filtered, and the filtrate was concentrated. The crude product was dissolved in dichloromethane and washed with water (2 × 25 mL), then the water washings were extracted with dichloromethane (20 mL), and the organic phases were combined. This combined organic phase was dried over Na_2_SO_4_ and concentrated in vacuum affording 2.16 g of crude product. This was purified by column chromatography on a silica gel bed (45 g). Gradient elution was used CH_2_Cl_2_ → CH_2_Cl_2_-MeOH 100:8.

Yield: 69% (1.43 g), brown, viscous oil. ${\left[\alpha \right]}_{D}^{25}$ = +63.1 (*c* = 1, CHCl_3_).

^1^H NMR (300 MHz, CDCl_3_) δ 4.82 (d, *J* = 3.5 Hz, 1H, H-1); 4.16–4.08 (m, 1H, H-6a); 3.85–3.51 (m, 18H, H-3, H-4, H-5, H-6b, 7 x OC*H*_2_); 3.50–3.43 (m, 1H, H-2); 3.43–3.33 (m, 7H, 2 x OC*H*_2_, OC*H*_3_); 3.30 (s, 3H, CH_2_OC*H*_3_); 2.89–2.50 (m, 6H, 3 x NC*H*_2_); 1.78–1.70 (m, 2H, CH_2_C*H*_2_CH_2_); 1.20 (t, *J* = 7.0 Hz, 3H, CH_2_C*H*_3_); 1.16 (t, *J* = 7.0 Hz, 3H, CH_2_C*H*_3_); ^13^C NMR (300 MHz. CDCl_3_) δ 97.52 (*C-1*); 81.82 (*C*H); 80.38 (*C*H); 77.85 (*C*H); 72.68 (*C*H); 70.82 (2 x O*C*H_2_CH_2_); 70.33 (2 x O*C*H_2_CH_2_); 70.29 (2 x O*C*H_2_CH_2_); 69.07 (*C*H_2_CH_3_); 68.43 (*C*H_2_CH_3_); 67.07 (O*C*H_2_CH); 58.81 (*C*H_2_OCH_3_); 55.17 (2 x O*C*H_3_); 53.74 (3x N*C*H_2_); 29.90 (NCH_2_*C*H_2_CH_2_OCH_3_); 16.06 (CH_2_*C*H_3_); 15.33 (CH_2_*C*H_3_).

HRMS calculated for C_23_H_45_NO_9_ 479.3094, found 479.3095.

#### 3.2.7. Methyl-4,6-di-*O*-ethyl-2,3-dideoxy-α-d-glucopyranosido[2,3-h]-*N*-[2-(2-methoxyphenyl)ethyl]-1,4,7,10-tetraoxa-13-azacyclopentadecane (**1c**)

Methyl-4,6-di-*O*-ethyl-2,3-bis-*O*-[(2-iodoethoxy)ethyl]-α-d-glucopyranoside (**9**) (2.80 g, 4.33 mmol) was dissolved in dry acetonitrile (60 mL), then 2-(2-methoxyphenyl)ethylamine (0.66 g, 4.33 mmol) and Na_2_CO_3_ (2.76 g, 26.0 mmol) were added. The mixture was refluxed under Ar atmosphere for 40 h. Upon completion of the reaction, the mixture was filtered, and the filtrate was concentrated. The crude product was dissolved in dichloromethane and washed with water (2 × 25 mL), then the water washings were extracted with dichloromethane (20 mL), and the organic phases were combined. This combined organic phase was dried over Na_2_SO_4_ and concentrated in vacuum affording 2.59 g of crude product. This was purified by column chromatography on a silica gel bed (77 g). Gradient elution was used CH_2_Cl_2_ → CH_2_Cl_2_-MeOH 100:4.

Yield: 60% (1.39 g), brown, viscous oil. ${\left[\alpha \right]}_{D}^{25}$ = +54.8 (*c* = 1, CHCl_3_).

^1^H NMR (300 MHz, CDCl_3_) δ 7.22–7.11 (m, 2H, Ar*H*); 6.90–6.80 (m, 2H, Ar*H*); 4.83 (d, *J* = 3.4 Hz, 1H, H-1); 4.12 (t, *J* = 9.4 Hz, 1H, H-6a); 3.85–3.52 (m, 21H, H-3, H-4, H-5, H-6b, ArOC*H*_3_, 7 x OC*H*_2_); 3.52–3.43 (m, 1H, H-2); 3.43–3.33 (m, 5H, OC*H*_2_, OC*H*_3_); 2.92–2.51 (m, 8H, 3 x NC*H*_2_, ArC*H*_2_); 1.20 (t, *J* = 7.0 Hz, 3H, CH_2_C*H*_3_); 1.17 (t, *J* = 7.0 Hz, 3H, CH_2_C*H*_3_); ^13^C NMR (300 MHz. CDCl_3_) δ 157.41 (Ar*C*OCH_3_); 130.54 (Ar*C*); 129.66 (Ar*C*); 123.94 (Ar*C*); 120.62 (Ar*C*); 110.28 (Ar*C*); 97.27 (*C-1*); 81.55 (*C*H); 80.08 (*C*H); 77.74 (*C*H); 72.49 (*C*H); 70.83 (2 x O*C*H_2_CH_2_); 70.17 (2 x O*C*H_2_CH_2_); 70.15 (2 x O*C*H_2_CH_2_); 70.03 (*C*H_2_CH_3_); 68.85 (*C*H_2_CH_3_); 68.23 (O*C*H_2_CH); 66.87 (N*C*H_2_); 55.30 (2 x O*C*H_3_); 54.95 (2x N*C*H_2_); 29.70 (NCH_2_*C*H_2_CH_2_OPhOCH_3_); 15.86 (CH_2_*C*H_3_); 15.13 (CH_2_*C*H_3_).

HRMS calculated for C_28_H_47_NO_9_ 541.3251 found 541.3255.

### 3.3. Asymmetric Reactions

#### 3.3.1. Synthesis of (2*R*,3*S*)-Phenyl(3-phenyloxirane-2-yl)methanone (**12**) via Darzens Condensation

2-Chloroacetophenone (0.15 g, 1 mmol) and benzaldehyde (0.15 mL, 1.5 mmol) and the appropriate crown catalyst (10 mol%) were dissolved in toluene (3 mL). Then 30% *aq.* NaOH solution (1 mL) was added, and the mixture was stirred at room temperature. The reaction was monitored by TLC (hexane—ethyl-acetate 10:1). After completing the reaction, the mixture was diluted with toluene (7 mL) and water (3 mL), and the phases were separated. The organic layer was washed with 10% *aq.* HCl solution (3 × 10 mL), dried (Na_2_CO_3_ and Na_2_SO_4_), filtered, and concentrated in vacuum. The crude product was purified by preparative TLC (hexane—ethyl-acetate 10:1) to give a yellowish-white powder with an mp of 64–66 °C. For the respective yields and *ee* values, see Table 1. Chiral HPLC: Phenomenex Lux^®^ 5u Cellulose-1 column, hexane:EtOH 85:15, major enantiomer t_R_ = 9.5 min, minor enantiomer t_R_ = 8.2 min. ${\left[\alpha \right]}_{D}^{22}$ = −132.7(*c* = 1, CH_2_Cl_2_) (62% ee)

^1^H NMR (CDCl_3_, 500 MHz), δ [ppm]: 7.97–7.94 (m, 2H, Ar*H*), 7.60–7.56 (m, 1H, Ar*H*), 7.46–7.44 (m, 2H, Ar*H*), 7.38–7.32 (m, 5H), 4.26 (d, *J* = 1.9 Hz, 1H, CO*CH*), 4.05 (d, *J* = 1.9 Hz, 1H, Ar*CH*); ^13^C NMR (75 MHz, CDCl_3_), δ [ppm]: 193.06 (*C*=O), 135.48 (Ar*C*), 133.97 (Ar*C*), 129.04 (Ar*C*), 128.86 (Ar*C*), 128.76 (Ar*C*), 128.33 (Ar*C*), 125.78 (Ar*C*), 61.00 (O*C*CO), 59.34 (Ph*C*O).

HRMS calculated for C_15_H_12_O_2_ 224.0837, found 224.0840.

#### 3.3.2. Synthesis of (2*R*,3*S*)-Phenyl(3-phenyloxirane-2-yl)methanone (**12**) via Epoxidation of *trans*-Chalcone

*trans*-Chalcone (0.25 g, 1.2 mmol) and the appropriate crown catalyst (10 mol%) were dissolved in toluene (3 mL), then 5.5 M *tert*-butylhydroperoxide solution (0.5 mL, in decane) and 20% *aq.* NaOH solution (1 mL) was added. The mixture was stirred at room temperature. The reaction was monitored by TLC (hexane—ethyl-acetate 10:1). After completion, the reaction mixture was diluted with toluene (7 mL) and water (3 mL), and the phases were separated. The organic layer was washed with 10% *aq.* HCl solution (3 × 10 mL), dried (Na_2_CO_3_ and Na_2_SO_4_), filtered, and concentrated in vacuum. The crude product was purified by preparative TLC (hexane—ethyl-acetate 10:1) to give a yellowish-white powder having an mp of 64–66 °C. For the respective yields and *ee* values, see Table 2. Chiral HPLC: Phenomenex Lux^®^ 5u Cellulose-1 column, hexane:EtOH 85:15, major enantiomer t_R_ = 8.2 min, minor enantiomer t_R_ = 9.4 min. ${\left[\alpha \right]}_{D}^{22}$ = −196.8 (*c* = 1, CH_2_Cl_2_) (92% ee).

^1^H NMR (CDCl_3_, 500 MHz), δ [ppm]: 7.97–7.94 (m, 2H, Ar*H*), 7.60–7.56 (m, 1H, Ar*H*), 7.46–7.44 (m, 2H, Ar*H*), 7.38–7.32 (m, 5H), 4.26 (d, *J* = 1.9 Hz, 1H, CO*CH*), 4.05 (d, *J* = 1.9 Hz, 1H, Ar*CH*); ^13^C NMR (75 MHz, CDCl_3_), δ [ppm]: 193.06 (*C*=O), 135.48 (Ar*C*), 133.97 (Ar*C*), 129.04 (Ar*C*), 128.86 (Ar*C*), 128.76 (Ar*C*), 128.33 (Ar*C*), 125.78 (Ar*C*), 61.00 (O*C*CO), 59.34 (Ph*C*O).

HRMS calculated for C_15_H_12_O_2_ 224.0837, found 224.0839.

#### 3.3.3. Synthesis of (*S*)-Diethyl 2-Acetamido-2-(2-nitro-1-phenylethyl)malonate (**16**) via Michael Addition

Diethyl acetamidomalonate (1.5 mmol), β-nitrostyrene (1.0 mmol), and the appropriate crown catalyst (10 mol%) were dissolved in a 4:1 mixture of dry diethyl ether and dry THF. After a short period of stirring, anhydrous Na_2_CO_3_ (0.20 g, 1.9 mmol) was added, and the mixture was stirred at room temperature. The reaction was monitored by TLC (hexane–ethyl-acetate 5:1). After completion, the solvents were removed in vacuum; the residue was dissolved in dichloromethane and filtered. The filtrate was washed with 10% *aq* HCl (3 × 10 mL) and dried (Na_2_CO_3_ and Na_2_SO_4_). The crude product obtained after evaporating the solvent was purified by preparative TLC (hexane—ethyl-acetate 10:1) to give an off-white solid having an mp of 135–136 °C. For the respective yields and *ee* values, see Table 3. Chiral HPLC: Phenomenex Lux^®^ 5u Amylose-2 column, hexane:EtOH 85:15, major enantiomer t_R_ = 16.8 min, minor enantiomer t_R_ = 18.6 min. = −42.8 (*c* = 1, CHCl_3_) (99% ee).

^1^H NMR (500 MHz, CDCl_3_) δ (ppm): 7.31–7.28 (m, 3H, Ar*H*), 7.22–7.18 (m, 2H, Ar*H*), 6.89 (br s, N*H*), 5.54–5.48 (m, 1H, PhC*H*), 4.73–4.66 (m, 2H, OC*H*_2_), 4.34–4.23 (m, 2H, OC*H*_2_), 4.20–4.13 (m, 1H, C*H*_2_NO_2_), 4.08–4.01 (m, 1H, C*H*_2_NO_2_), 2.12 (s, 3H, COC*H*_3_), 1.27 (t, *J* = 7 Hz, 3H, C*H*_3_CH_2_), 1.25 (t, *J* = 7 Hz, 3H, C*H*_3_CH_2_); ^13^C NMR (75 MHz, CDCl_3_), δ [ppm]: 170.10 (*C*OCH_3_), 166.43 (*C*(O)O), 165.71 (*C*(O)O), 133.78 (Ar*C*), 128.75 (Ar*C*), 128.70 (Ar*C*), 128.69 (Ar*C*), 76.83 (HN*C*CO), 67.21 (*C*NO_2_), 63.56 (*C*H_2_CH_3_), 62.75 (*C*H_2_CH_3_), 48.30 (Ph*C*CNO_2_), 22.97 (CO*C*H_3_), 13.84 (CH_2_*C*H_3_), 13.76 (CH_2_*C*H_3_).

HRMS calculated for C_17_H_22_N_2_O_7_ 366.1727, found 366.1728.

#### 3.3.4. Synthesis of (*R*)-Diethyl 2,2-Dicyano-3-phenylcyclopropane-1,1-dicarboxylate (**19**) via MIRC Reaction

Benzylidenemalononitrile (1.0 mmol), diethyl bromomalonate (1.5 mmol), and the appropriate crown ether (10 mol%) were dissolved in anhydrous CH_2_Cl_2_ (3 mL), and dry Na_2_CO_3_ (2.0 mmol) was added. The reaction mixture was stirred at room temperature. After completing the reaction, the mixture was filtered, then the organic phase was washed with 10% *aq.* HCl (3 × 10 mL) and then with water (10 mL), dried (Na_2_CO_3_ and Na_2_SO_4_) and concentrated. The crude product was purified by preparative TLC using hexane–ethyl acetate (5:1) as the eluent to give a yellow oil. For the respective yields and *ee* values, see Table 4. Chiral HPLC: Kromasil 5-Amycoat^®^ column, hexane:EtOH 85:15, major enantiomer t_R_ = 8.7 min, minor enantiomer t_R_ = 7.4 min. ${\left[\alpha \right]}_{D}^{22}$ = −20.3 (*c* = 1, CHCl_3_) (99% ee).

^1^H NMR (300 MHz, CDCl_3_),δ (ppm): 7.45–7.35 (m, 5H, Ar*H*), 4.43 (q, *J* = 7.2 Hz, 2H, OC*H*_2_), 4.30–4.18 (m, 2H, OC*H*_2_), 3.96 (s, 1H, ArC*H*), 1.39 (t, *J* = 7.2 Hz, 3H, CH_2_C*H*_3_), 1.19 (t, *J* = 7.2 Hz, 3H, CH_2_C*H*_3_); ^13^C NMR (75 MHz, CDCl_3_), δ (ppm): 163.05 (*C*OOC_2_H_5_), 161.06 (*C*OOC_2_H_5_), 129.67 (*ArC*), 129.10 (*ArC*), 128.76 (*ArC*), 127.31 (*ArC*), 111.86 (*C*N), 109.71 (*C*N), 64.50 (*C*H_2_CH_3_), 63.62 (*C*H_2_CH_3_), 46.39 (OC*C*CO), 40.08 (Ph*C*H), 16.32 (NC*C*CN), 13.97 (CH_2_*C*H_3_), 13.60 (CH_2_*C*H_3_).

HRMS calculated for C_17_H_16_N_2_O_4_ 312.1110 found 312.1112.

## 4. Conclusions

New chiral crown ethers annulated to methyl 4,6-di-*O*-ethyl-α-d-glucopyranoside (**1a**–**c**) have been synthesized and tested in asymmetric reactions as phase transfer catalysts. Their effectiveness was compared to their 4,6-*O*-benzylidene analogues (**2a**–**c**). It was found that the absence of the two-ring annulation affects the enantioselectivity rather negatively. Still, the results suggest that the effects of the protecting group(s) attached to the oxygen atoms in positions 4 and 6 of the carbohydrate and that of the sidearm are not independent of each other.

In the liquid–liquid model reactions, lariat ethers with a hydroxypropyl side chain were the most effective as observed to date. In the case of Michael addition of diethyl acetamidomalonate, the same phenomenon was experienced, which suggests that the interaction of the OH group has a crucial role in the formation of enantioselectivity. However, in the MIRC reaction of benzylidenemalononitrile and diethyl bromomalonate, the methoxypropyl side arm proved to be more effective. In addition, the 4,6-di-*O*-ethyl-α-d-glucopyranoside-based crown catalyst (**1b**) was superior to its 4,6-*O*-benzylidene analogue (**2b**) in this cyclopropanation reaction. In this case, better flexibility was beneficial to the asymmetric induction.

Since the new 4,6-di-*O*-ethyl-glucoside-based crown ethers do not contain acid-sensitive groups, they may be suitable for recovery through salt formation by extraction with mineral acid without any kind of structural alteration. By changing the 4,6 protecting groups of the glucose unit, lipophilicity and thus recoverability can be affected. Attempts to recover and reuse this type of chiral macrocycles are ongoing in our research group.

## Figures and Tables

**Table 1:** Effect of the crown catalysts (**1** or **2**) in Darzens condensation of 2-chloroacetophenone (**10**) and benzaldehyde (**11**).

Entry	Catalyst	Time, h	Yield, % ^a^	ee, % ^b^
1	**1a**	1	69	52
2	**1b**	1	68	29
3	**1c**	1	66	19
4	**2a**	1	74	62 ^c^
5	**2b**	1	74	21 ^c^
6	**2c**	1	38	29

^a^ Isolated by preparative TLC; ^b^ Determined by chiral HPLC; ^c^ See Ref. [63].

**Table 2:** Effect of the crown catalysts (**1** or **2**) in the epoxidation of chalcone (**13**).

Entry	Catalyst	Time, h	Yield, % ^a^	ee, % ^b^
1	**1a**	4	79	75
2	**1b**	4	87	24
3	**1c**	24	70	21
4	**2a**	1	82	92 ^c^
5	**2b**	2	61	23 ^c^
6	**2c**	72	73	3

^a^ Isolated by preparative TLC; ^b^ Determined by chiral HPLC; ^c^ See Ref. [64].

**Table 3:** Effect of the glucose-based macrocycles (**1** or **2**) in Michael addition of β-nitrostyrene (**14**) and diethyl acetamidomalonate (**15**).

Entry	Catalyst	Time, h	Yield, % ^a^	ee, % ^b^
1	**1a**	72	49	42
2	**1b**	48	42	28
3	**1c**	120	33	21
4	**2a**	3	70	99 ^c^
5	**2b**	22	58	38
6	**2c**	120	36	15

^a^ Isolated by preparative TLC; ^b^ Determined by chiral HPLC; ^c^ See Ref. [65].

**Table 4:** Effect of the monoaza macrocycles (**1** or **2**) in the MIRC reaction of benzylidenemalononitrile (**17**) with diethyl bromomalonate (**18**).

Entry	Catalyst	Time, h	Yield, % ^a^	ee, % ^b^
1	**1a**	48	38	22
2	**1b**	24	40	99
3	**1c**	24	92	15
4	**2a**	20	82	32 ^c^
5	**2b**	24	97	70
6	**2c**	24	81	58

^a^ Isolated by preparative TLC; ^b^ Determined by chiral HPLC; ^c^ See Ref. [66].

## Sample Availability

Samples of the compounds are not available from the authors.
